# Supplementary material for: A spatiotemporal transcriptomic atlas of porcine (Sus scrofa) female early gonadal development
Source: Commun Biol. 2026 Mar 30;9:487. doi: 10.1038/s42003-026-09932-0 (PMC13050328; doi:10.1038/s42003-026-09932-0)
Supplement: Supplementary file 1 — Supplementary Information [file 42003_2026_9932_MOESM1_ESM.pdf]

A spatiotemporal transcriptomic atlas of porcine (*Sus scrofa*) female early gonadal development

Authors

Pengcheng He[1,#], Wenzhe Xia[1,#], Tianzhi Chen[1,#], Yaxuan Yan[1,#], Dengfeng Gao[2,#], Yadi Teng[1], Ting Zhao[1], Xinze Chen[1], Zhiqiang Feng[1], Runbo Li[1], Meng Wang[1], Yuwen Ke[1,*] & Jianyong Han[1,*]

Affiliations

[1]Frontiers Science Center for Molecular Design Breeding (MOE), State Key Laboratory of Animal Biotech Breeding, College of Biological Sciences, China Agricultural University, Beijing 100193, China.

[2]State Key Laboratory of Swine and Poultry Breeding Industry, College of Animal Science and Technology, Sichuan Agricultural University, Chengdu, Sichuan 611130, China.

[#]These authors contributed equally: Pengcheng He, Wenzhe Xia, Tianzhi Chen, Yaxuan Yan and Dengfeng Gao.

[*]Correspondence should be addressed to Y.K.(email: keyw@cau.edu.cn), J.H.(email: hanjy@cau.edu.cn)

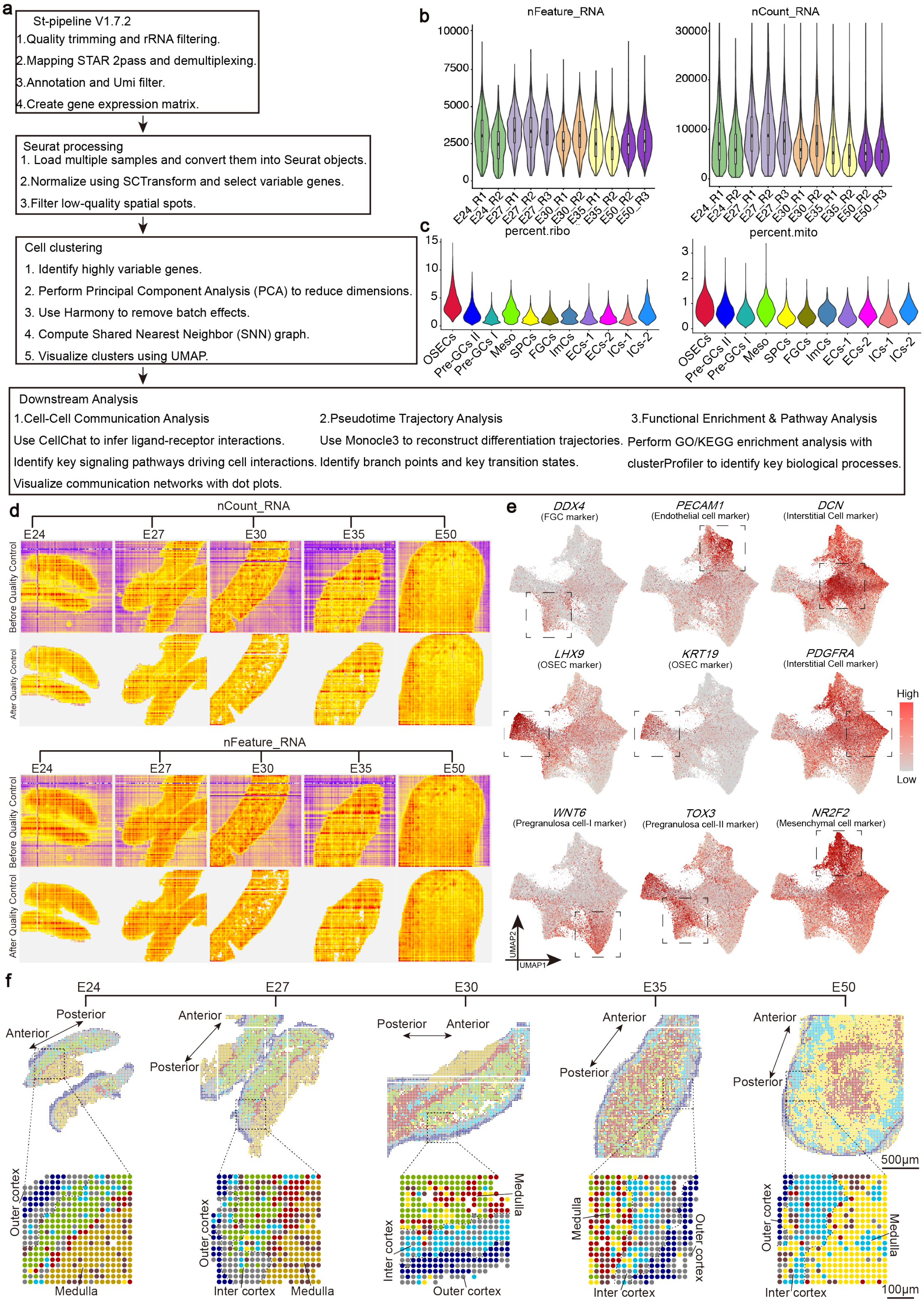

Supplementary Figure 1: Quality control of porcine female gonad spatial transcriptome data. a, The schematic illustrates the pipeline for analyzing high-resolution spatial transcriptomic data, including data preprocessing, quality control, and downstream analyses. **b-d,** Data quality control for RNA. nCount_RNA: Total RNA

(UMI) count per cell. It represents the sum of the transcript numbers (Unique Molecular Identifiers, UMIs) of all genes within the cell. A value that is too high may indicate doublets, and a value that is too low may suggest a low-quality cell. nFeature_RNA: Number of genes detected per cell. It represents the number of different genes (features) expressed in the cell. A value that is too low may imply poor cell quality (such as lysed cells), and a value that is too high may indicate doublets. **e,** UMAP visualization of representative genes for female fetal germ cells and gonadal somatic cells. Expression levels were color coded. **f,** Spatial mapping of cell populations in replicate samples. Cellular distributions were reconstituted on the microarray coordinate system through HDst-seq barcode alignment. The dashed box indicates the enlarged view of the corresponding area. The outer cortical, inner cortical, and medullary regions of the gonads are delineated by dashed lines. Scale bar: 500 μm (zoomed-out), 100 μm (zoomed-in).

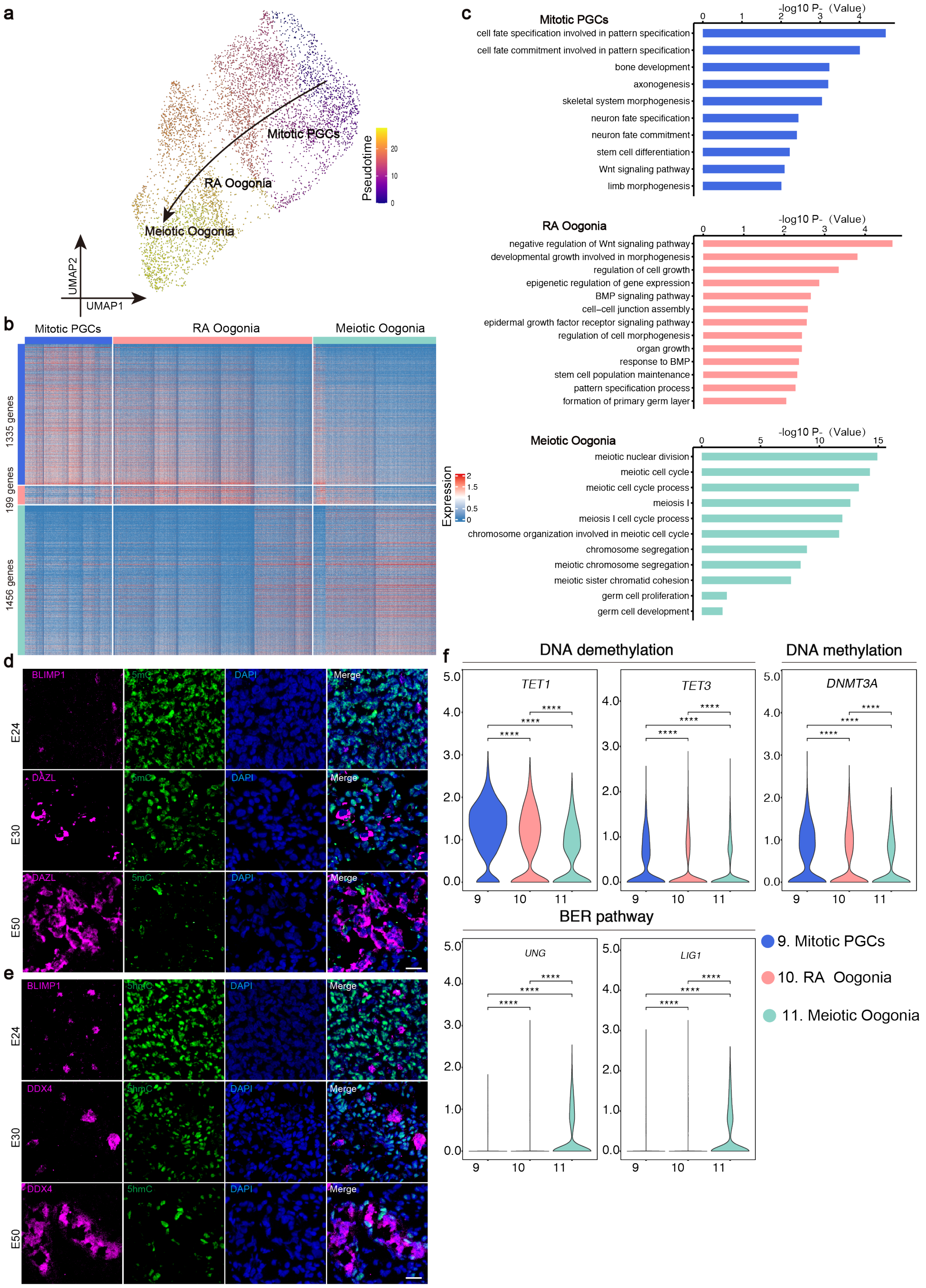

Supplementary Figure 2: Molecular profiling and developmental dynamics of three FGC subtypes. a, Monocle3 was employed to conduct UMAP dimensionality reduction analysis on the spatial transcriptome data of three types of FGCs, and the cell development pathway was revealed through pseudotime trajectory Reconstitution. **b,**

Heatmap depicting differentially expressed genes across three female germ cell stages, with color gradient from blue (low expression) to red (high expression). **c,** GO enrichment analysis revealed the characteristic biological processes of each germ cell subpopulation. The height of the bar chart indicates the enrichment significance. **d,** Immunofluorescence images of BLIMP1/DAZL (purple) and 5mC (green) in porcine female gonadal sections from different timepoints. Nuclei were counterstained with DAPI (blue). Scale bar, 50 μm. **e,** Immunofluorescence images of BLIMP1/DDX4 (purple) and 5hmC (green) in porcine female gonadal sections from different timepoints. Nuclei were counterstained with DAPI (blue). Scale bar, 50 μm. **f,** Violin plots showing expression of epigenetic modifiers for DNA demethylation, DNA methylation and BER pathway components. Results are analyzed by wilcox test. *adjp < 0.05; **adjp < 0.01; ***adjp < 0.001; ****adjp < 0.0001; ns, not significant, adjP ≥ 0.05.

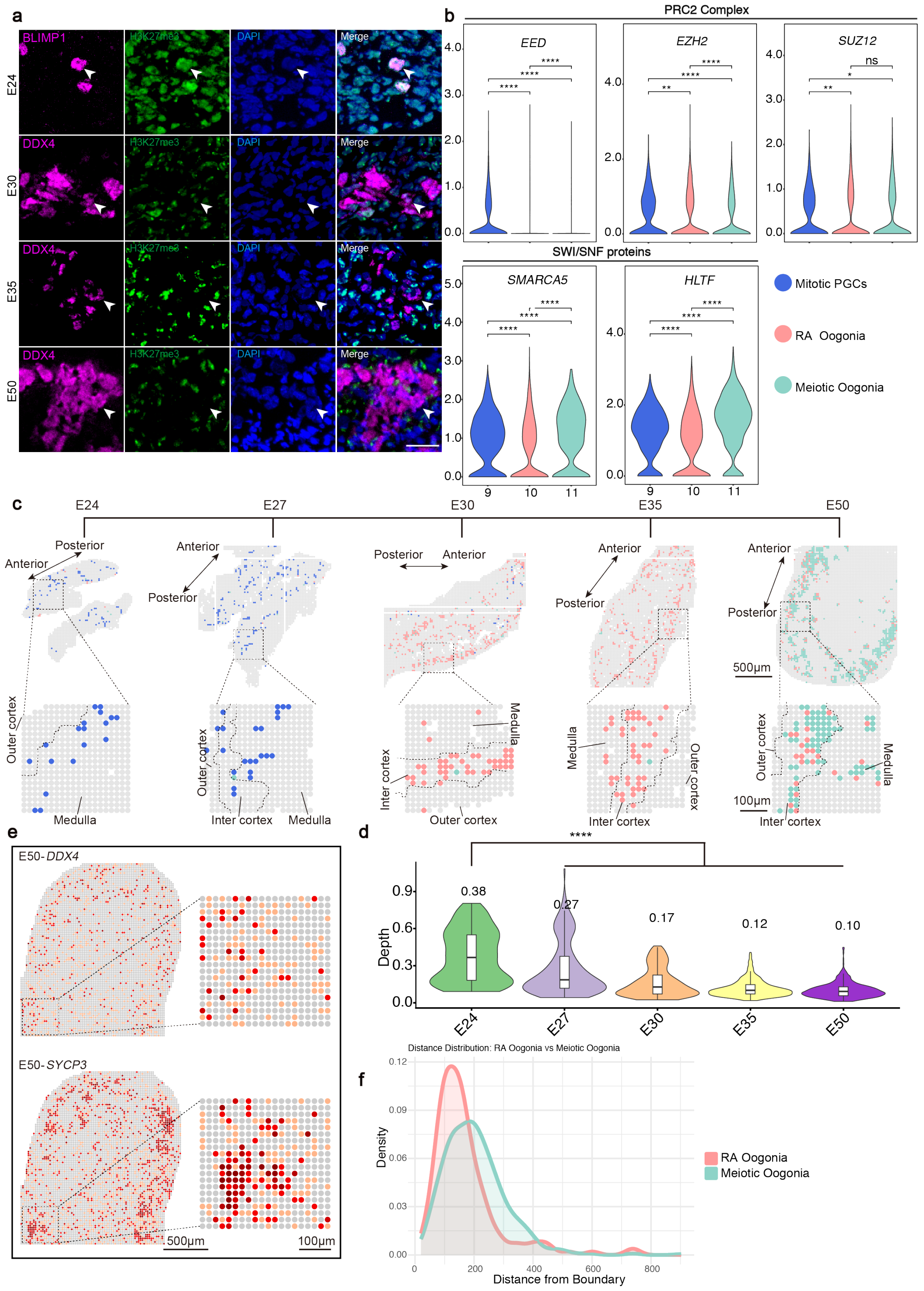

Supplementary Figure 3: Epigenetic dynamics during pPGC development. a, Immunofluorescence images of DDX4 (green) and H3K27me3 (purple) in porcine female gonadal sections from different timepoints (E24, E30, E35, and E50). Nuclei were counterstained with DAPI (blue). At E24, germ cells show co-localization of

BLIMP1 and H3K27me3; at E30, only a very small number of DDX4-positive germ cells exhibit co-localization with H3K27me3; and at E35 and E50, no co-localization is observed between DDX4 and H3K27me3 in germ cells. Scale bar, 50 μm. **b,** Violin plots showing expression of epigenetic modifiers for PRC2 Complex and SWI/SNF proteins. Results are analyzed by wilcox test. *adjp < 0.05; **adjp < 0.01; ***adjp < 0.001; ****adjp < 0.0001; ns, not significant, adjP ⩾ 0.05. **c,** Spatial localization of three distinct subgroups of porcine FGCs is shown in representative images from replicate samples. Germ cells at different stages are color-coded as indicated. The outer cortical, inner cortical, and medullary regions of the gonads are delineated by dashed lines. The dashed box indicates the enlarged view of the corresponding area. Scale bar: 500 μm (zoomed-out), 100 μm (zoomed-in). **d,** Statistical analysis the distance between germ cells and the outermost cortex during different periods. **e,** Spatial visualization of specifically expressed genes at E50. The dashed box indicates the enlarged view of the corresponding area. Scale bar: 500 μm (zoomed-out), 100 μm (zoomed-in). **f,** Kernel density plots showing the spatial distance of two fetal germ cell subpopulations (RA Oogonia and Meiotic Oogonia) from a defined tissue boundary in the E50 female gonad. The x-axis represents the Distance from Boundary (in μm), and the y-axis represents the Density (probability distribution).

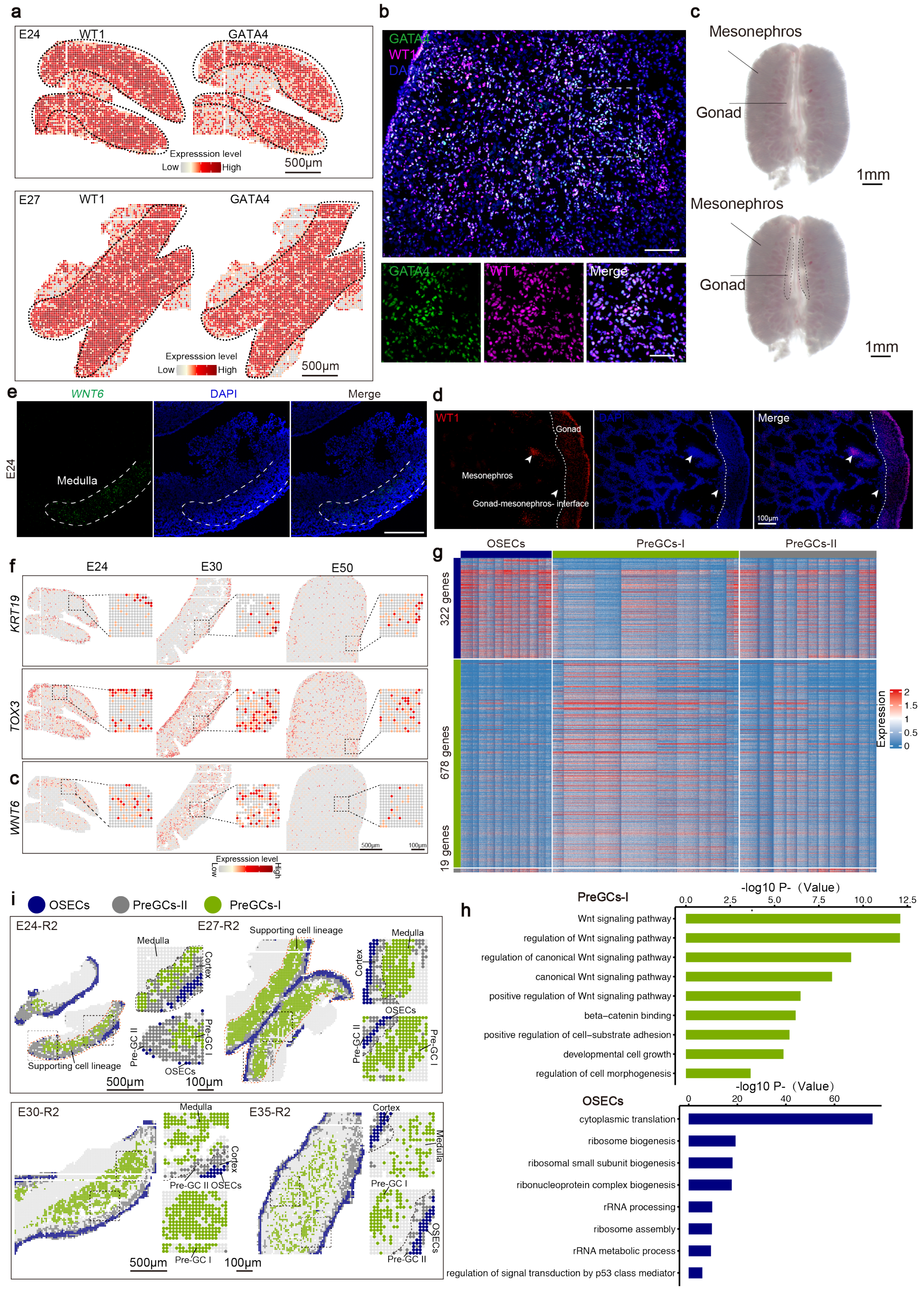

Supplementary Figure 4: Spatiotemporal dynamics of the supporting cell lineage in porcine female gonadal development. a, Spatial visualization shows the dynamic expression patterns of *GATA4* and *WT1* at embryonic stages E24 and E27. The gene expression levels are color-coded, with a redder color indicating a higher expression

level. Scale bar: 500 µm. **b,** Immunofluorescence images of GATA4 (green) and WT1 (purple) in porcine female gonadal sections from E50 gonad. The cell nuclei were counterstained with DAPI (blue). The dashed box indicates the enlarged view of the corresponding area. Scale bar: 100 µm (zoomed-out), 50 µm (zoomed-in). **c,** Bright-field microscopy of E24 gonadal-mesonephric tissue. The black dashed line denotes the gonads. Scale bars, 1 mm. **d,** Immunofluorescence images of WT1 (red) in porcine female E24 gonadal sections. Nuclei were counterstained with DAPI (blue). Scale bar, 100 µm. The dotted line indicates the demarcation between the gonad and the mesonephros; the left side shows the mesonephros, and the right side shows the gonad. The left arrow indicates WT-positive cells, and the right arrow indicates WT-negative cells. **e,** RNA in situ hybridization for *WNT6* (green, probe) in porcine female gonadal sections from E24. The dashed area indicates the enrichment zone of *WNT6*-positive cells. The cell nuclei were counterstained with DAPI (blue). Scale bar: 250 µm. **f,** Spatial transcriptomic mapping of lineage-specific marker genes across the three supporting cell subtypes. The dashed box indicates the enlarged view of the corresponding area. Scale bar: 500 µm (zoomed-out), 100 µm (zoomed-in). **g,** Heatmap of differentially expressed genes defining the three supporting cell subtypes. The color key from blue to red indicates gene expression levels from low to high respectively. **h,** GO enrichment analysis showing the characteristic biological processes of OSECs and PreGCs-I. The height of the bar chart indicates the enrichment significance. **i,** Spatial visualization showing localization of three supporting cell lineage subsets across different timepoints. The three cell types of supporting cell lineage were represented by different colors. Panels (a) and (b) show magnified views of different regions in the figure. The cortical and medullary regions of the gonads are delineated by dashed lines. Scale bars: 500 µm (zoomed-out), 100 µm (zoomed-in).

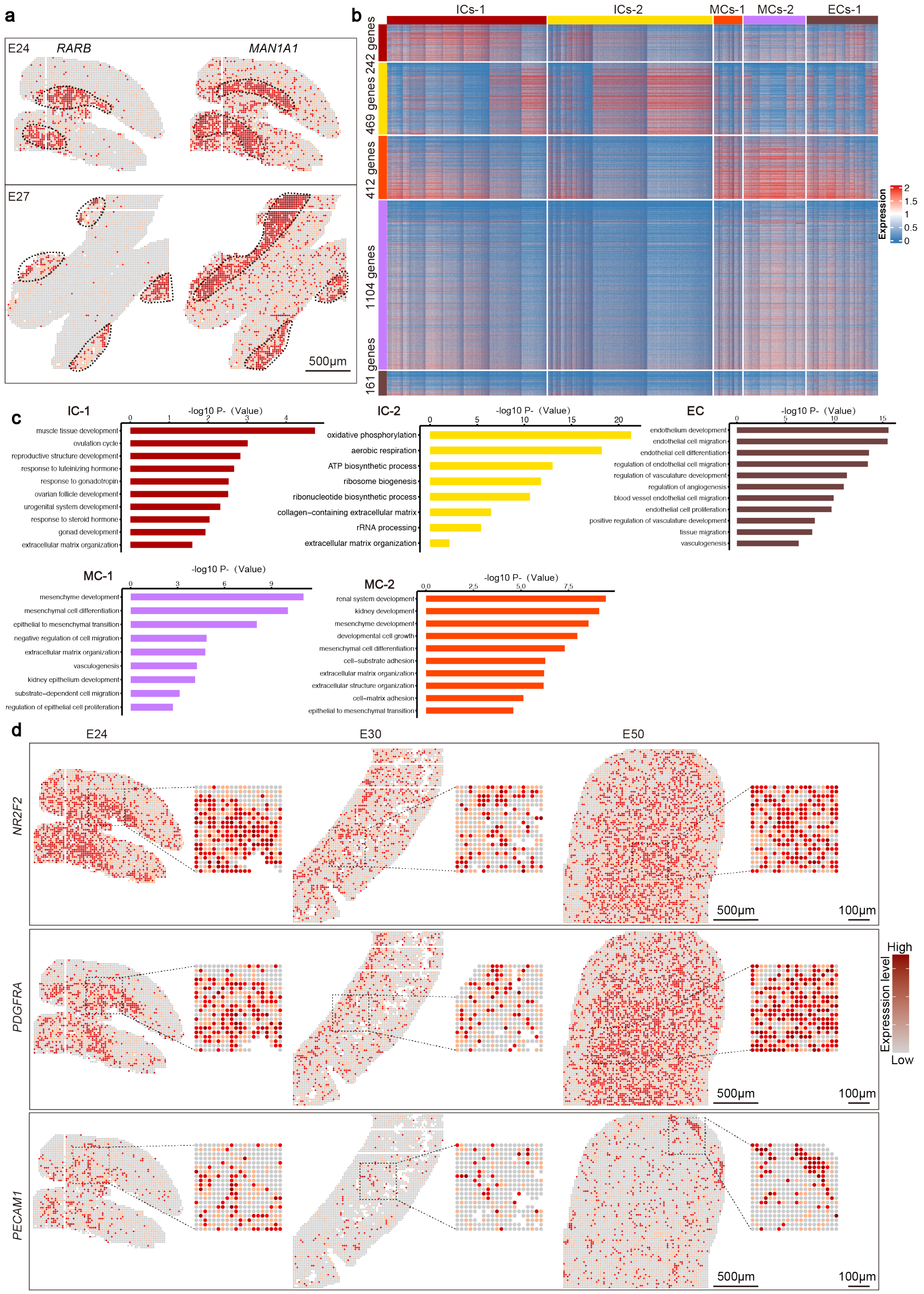

Supplementary Figure 5: Spatial localization of ECs, ICs, and SPCs. a, Spatial visualization showing spatial localization of *RARB* and *MAN1A1* in E24 and E27 stages. The red gradient indicates the expression abundance. The dashed lines indicate the enriched regions of *RARB/MAN1A1* genes. Scale bar: 500 μm. **b,** Heatmap of

differentially expressed genes defining the interstitial cells, mesenchymal cells and endothelial cells. The color key from blue to red indicates gene expression levels from low to high respectively. **c,** GO enrichment analysis showing the subgroup-specific biological processes. The height of the bar chart indicates the enrichment significance. **d,** Spatial visualization of specifically expressed genes across interstitial cells, endothelial cells and mesenchymal cells. The dashed box indicates the enlarged view of the corresponding area. Scale bars, 500 μm (zoomed-out), 100 μm (zoomed-in).

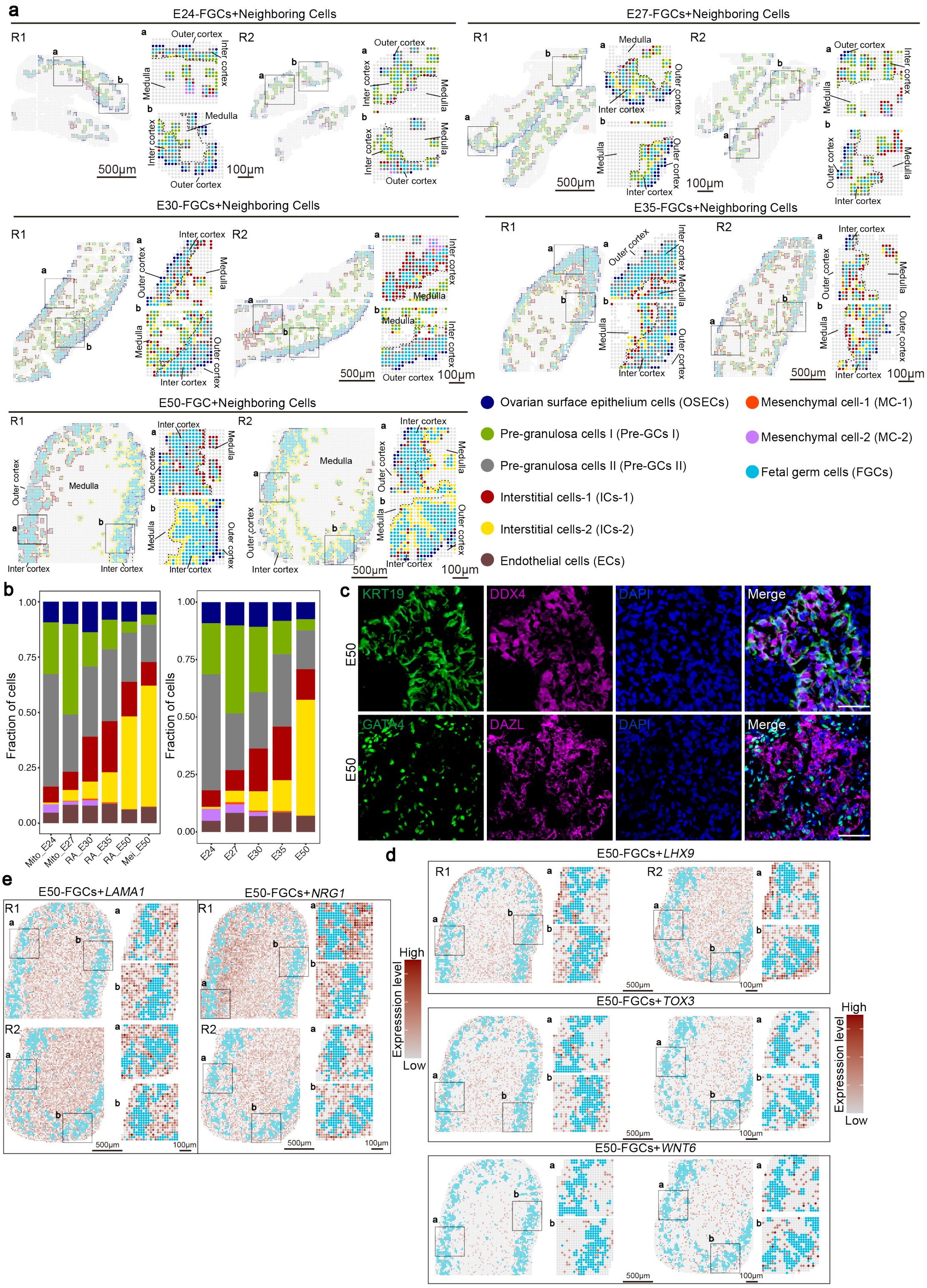

Supplementary Figure 6: Spatiotemporal dynamics of FGCs and niche during porcine gonadal development. a, Spatial visualization showing FGCs surrounding niches across developmental stages from E24 to E50. Panels (a) and (b) show magnified views of different regions in the figure. The outer cortical, inner cortical, and medullary

regions of the gonads are delineated by dashed lines. Scale bars: 500 μm (zoomed-out), 100 μm (zoomed-in). **b,** Bar chart shows the distribution proportions of different types of germ cells at different periods, colored according to different cell types. **c,** Immunofluorescence images of KRT19 (green) and DDX4 (purple) in porcine female gonadal sections in E50 gonad. Nuclei were counterstained with DAPI (blue), in the top figure. Scale bar: 50 μm. Immunofluorescence images of GATA4 (green) and DDX4 (purple) in porcine female gonadal sections in E50 gonad. Nuclei were counterstained with DAPI (blue), in the bottom figure. Scale bar: 50 μm. **d,** Spatial visualization showing FGCs and gene localization in supporting cell lineages. Scale bars, 500 μm (zoomed-out), 100 μm (zoomed-in). **e,** Spatial visualization showing FGCs and gene localization in other cell types. Panels (a) and (b) show magnified views of different regions in the figure. Scale bars, 500 μm (zoomed-out), 100 μm (zoomed-in).

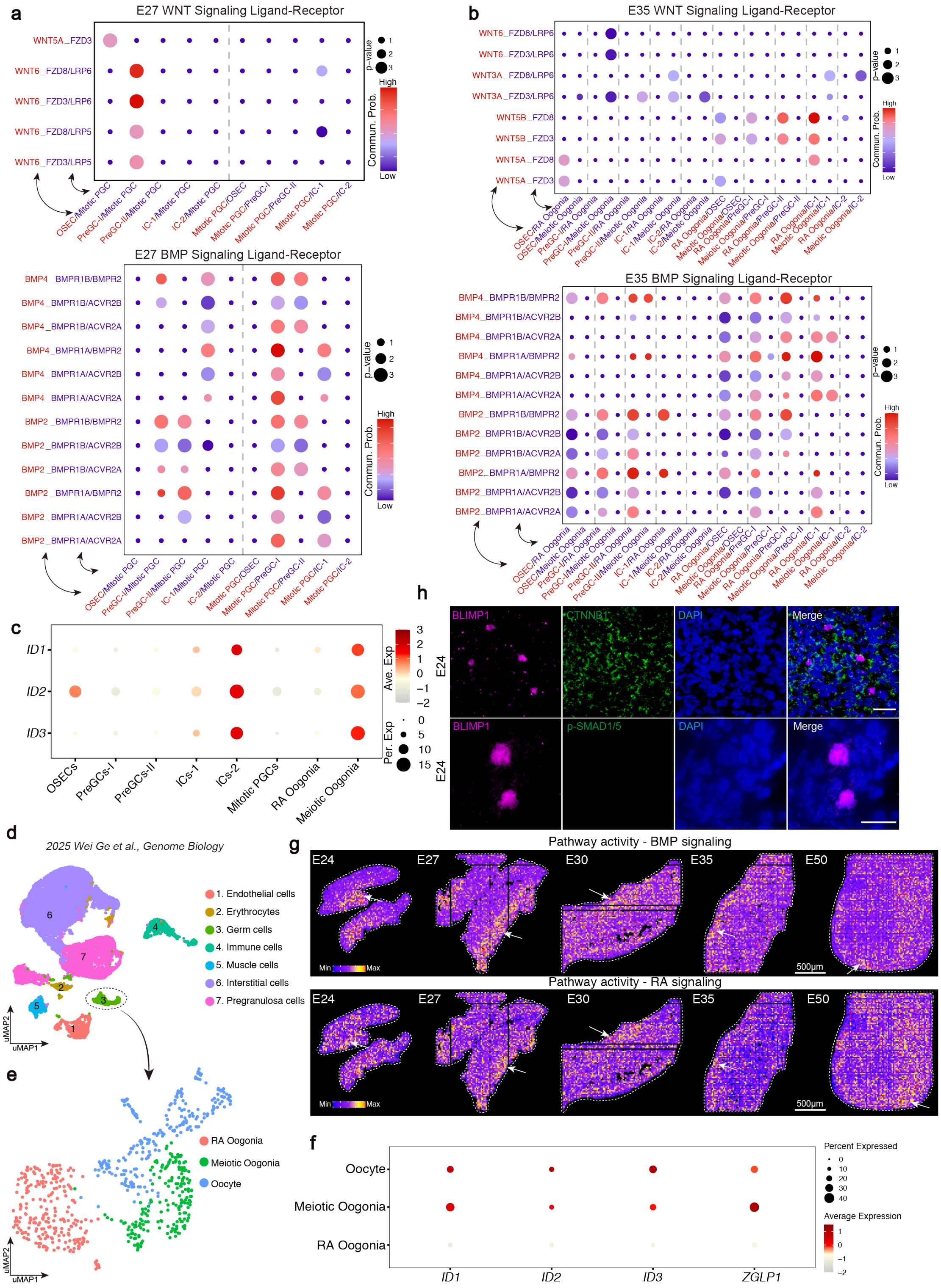

Supplementary Figure 7: Expression of ligand–receptor pairs and downstream pathway effectors in supporting cells of fetal female gonads. a, WNT and BMP Ligand-receptor pairs among fetal germ cells (Mitotic PGCs, RA Oogonia, Meiotic Oogonia), three supporting cell lineage subsets (OSECs, PreGCs-II, and PreGCs-I) and interstitial cells (IC-1 and IC-2) at E27. E27 WNT Ligand-receptor pairs (Top); E27

BMP Ligand-receptor pairs (Bottom). **b,** WNT and BMP Ligand-receptor pairs among fetal germ cells (Mitotic PGCs, RA Oogonia, Meiotic Oogonia), three supporting cell lineage subsets (OSECs, PreGCs-II, and PreGCs-I) and interstitial cells (IC-1 and IC-2) at E35. E35 WNT Ligand-receptor pairs (Top); E35 BMP Ligand-receptor pairs (Bottom). **c,** Dot plot shows the expression of BMP-related *ID* genes in FGCs. The dot diameter indicates the proportion of expressing cells, and the color gradient reflects the expression level. **d,** UMAP visualization of single-cell clustering: Distinct cell populations of germ and somatic cells in developing female porcine gonads. Dashed area indicates fetal germ cell cluster. **e,** UMAP analysis reveals three fetal germ cell subpopulations: RA Oogonia, Meiotic Oogonia and Oocyte. **f,** Dot plot shows the expression of BMP-related *ID* genes and *ZGLP1* in FGCs. The dot diameter indicates the proportion of expressing cells, and the color gradient reflects the expression level. **g,** Representative images of the spatial activity of the BMP and RA pathways in the developing female pig gonad at indicated time points. The white arrows indicate the enriched regions of BMP/RA signaling activation. Max, maximum; min, minimum. Scale bar: 500 μm. **h,** Immunofluorescence images of BLIMP1 (purple) and CTNNB1 (green) in porcine female gonadal sections from different timepoints. Nuclei were counterstained with DAPI (blue), in the top figure. Scale bars: 20 μm. Immunofluorescence images of BLIMP1 (purple) and p-SMAD1/5 (green) in porcine female gonadal sections from different timepoints. Nuclei were counterstained with DAPI (blue), in the bottom figure. Scale bars: 20 μm.

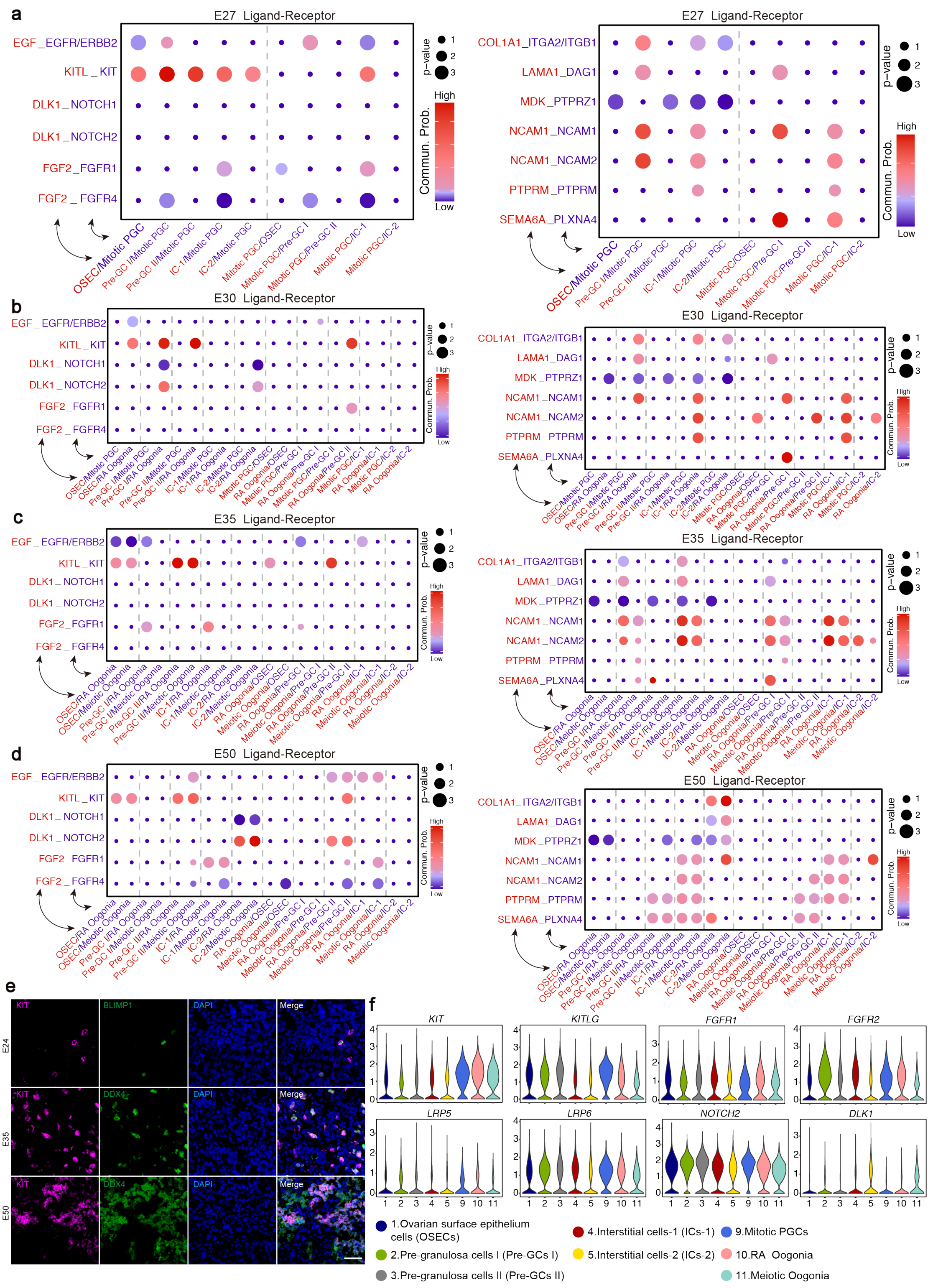

Supplementary Figure 8. Ligand-receptor pairs between FGC and other gonadal somatic cells.

a, RTK and other Ligand-receptor pairs among fetal germ cells (Mitotic PGCs, RA Oogonia, Meiotic Oogonia), three supporting cell lineage subsets (OSECs, PreGCs-II,

and PreGCs-I) and interstitial cells (IC-1 and IC-2) at E27. E27 RTK Ligand-receptor pairs (left); E27 other Ligand-receptor pairs (right). **b,** RTK and other Ligand-receptor pairs among fetal germ cells three supporting cell lineage subsets and interstitial cells at E30. E30 RTK Ligand-receptor pairs (left); E30 other Ligand-receptor pairs (right). **c,** RTK and other Ligand-receptor pairs among fetal germ cells three supporting cell lineage subsets and interstitial cells at E35. E35 RTK Ligand-receptor pairs (left); E35 other Ligand-receptor pairs (right). **d,** RTK and other Ligand-receptor pairs among fetal germ cells three supporting cell lineage subsets and interstitial cells at E50. E50 RTK Ligand-receptor pairs (left); E50 other Ligand-receptor pairs (right). **e,** Immunofluorescence images of KIT (purple) and DDX4 (green) in porcine female gonadal sections from different timepoints. Nuclei were counterstained with DAPI (blue), Scale bar: 50 μm. **f,** Violin plots of selected RTK-associated ligands and receptors in female germ and somatic clusters.
