# Supplementary material for: A spatiotemporal transcriptomic atlas of porcine (Sus scrofa) female early gonadal development
Source: Commun Biol. 2026 Mar 30;9:487. doi: 10.1038/s42003-026-09932-0 (PMC13050328; doi:10.1038/s42003-026-09932-0)
Supplement: Supplementary file 2 — Description of Additional Supplementary Files [file 42003_2026_9932_MOESM2_ESM.pdf]

Description of Additional Supplementary files

File name: Supplementary Data 1

Description: Metadata of spatial transcriptomics datasets and the list of differentially expressed genes identified in this study.

File name: Supplementary Data 2

Description: Differentially expressed genes identified in germ cell lineage populations, together with Gene Ontology (GO) enrichment results for cluster-specific upregulated genes.

File name: Supplementary Data 3

Description: Differential expression analysis of epigenetic modification-related genes in germ cell populations.

File name: Supplementary Data 4

Description: Differentially expressed genes identified in supporting cell lineage populations, together with Gene Ontology (GO) enrichment results for cluster-specific upregulated genes.

File name: Supplementary Data 5

Description: Differentially expressed genes associated with interstitial and mesenchymal cell lineage populations and Gene Ontology (GO) enrichment analysis of upregulated genes in each cluster.

File name: Supplementary Data 6

Description: Comprehensive summary of cell–cell communication interactions between germ cells and surrounding neighboring cells (within one spatial grid) across different developmental stages.
