# Supplementary material for: A spatiotemporal transcriptomic atlas of porcine (Sus scrofa) female early gonadal development
Source: Commun Biol. 2026 Mar 30;9:487. doi: 10.1038/s42003-026-09932-0 (PMC13050328; doi:10.1038/s42003-026-09932-0)
Supplement: Supplementary file 9 — Reporting Summary [file 42003_2026_9932_MOESM9_ESM.pdf]

nature portfolio

Corresponding author(s): *Double-anonymous peer review submissions: write DAPR and your manuscript number here instead of author names.*

Last updated by author(s): *YYYY-MM-DD*

Reporting Summary

Nature Portfolio wishes to improve the reproducibility of the work that we publish. This form provides structure for consistency and transparency in reporting. For further information on Nature Portfolio policies, see our Editorial Policies and the Editorial Policy Checklist.

Statistics

For all statistical analyses, confirm that the following items are present in the figure legend, table legend, main text, or Methods section.

n/a	Confirmed	
☐	☒	The exact sample size (*n*) for each experimental group/condition, given as a discrete number and unit of measurement
☐	☒	A statement on whether measurements were taken from distinct samples or whether the same sample was measured repeatedly
☐	☒	The statistical test(s) used AND whether they are one- or two-sided<br>*Only common tests should be described solely by name; describe more complex techniques in the Methods section.*
☐	☒	A description of all covariates tested
☒	☐	A description of any assumptions or corrections, such as tests of normality and adjustment for multiple comparisons
☐	☒	A full description of the statistical parameters including central tendency (e.g. means) or other basic estimates (e.g. regression coefficient) AND variation (e.g. standard deviation) or associated estimates of uncertainty (e.g. confidence intervals)
☐	☒	For null hypothesis testing, the test statistic (e.g. *F*, *t*, *r*) with confidence intervals, effect sizes, degrees of freedom and *P* value noted<br>*Give P values as exact values whenever suitable.*
☒	☐	For Bayesian analysis, information on the choice of priors and Markov chain Monte Carlo settings
☒	☐	For hierarchical and complex designs, identification of the appropriate level for tests and full reporting of outcomes
☒	☐	Estimates of effect sizes (e.g. Cohen's *d*, Pearson's *r*), indicating how they were calculated

Our web collection on statistics for biologists contains articles on many of the points above.

Software and code

Policy information about availability of computer code

Data collection

For spatial transcriptomics, the constructed libraries were sequenced on the Illumina NovaSeq 6000 platform using paired-end 150 bp reads.
Data analysis: ImageJ (Fiji, version 1.8.0, National Institute of Health, USA)
The data of fluorescence were collected by inverted microscopy (OLYMPUS, CKX41, Leica TCS SP8 STED 3X).

Data analysis

Cutadapt (v1.18)
Umi-tools (v1.1.0)
St-pipeline (v1.8.1)
Seurat (v4.4.0)
Harmony (v1.2.3)
Monocle3 (v1.3.1)
Clusterprofiler (v4.11.0)
Cellchat (v1.6.1)
ggplot2 (v3.1.0)

For manuscripts utilizing custom algorithms or software that are central to the research but not yet described in published literature, software must be made available to editors and reviewers. We strongly encourage code deposition in a community repository (e.g. GitHub). See the Nature Portfolio guidelines for submitting code & software for further information.

Data

Policy information about availability of data

All manuscripts must include a data availability statement. This statement should provide the following information, where applicable:

- Accession codes, unique identifiers, or web links for publicly available datasets
- A description of any restrictions on data availability
- For clinical datasets or third party data, please ensure that the statement adheres to our policy

Data are available in the main text, supplementary materials and Genome Sequence Archive (CRA023499).

Research involving human participants, their data, or biological material

Policy information about studies with human participants or human data. See also policy information about sex, gender (identity/presentation), and sexual orientation and race, ethnicity and racism.

Reporting on sex and gender	N/A
Reporting on race, ethnicity, or other socially relevant groupings	N/A
Population characteristics	N/A
Recruitment	N/A
Ethics oversight	N/A

Note that full information on the approval of the study protocol must also be provided in the manuscript.

Field-specific reporting

Please select the one below that is the best fit for your research. If you are not sure, read the appropriate sections before making your selection.

☒ Life sciences ☐ Behavioural & social sciences ☐ Ecological, evolutionary & environmental sciences

For a reference copy of the document with all sections, see nature.com/documents/nr-reporting-summary-flat.pdf

Life sciences study design

All studies must disclose on these points even when the disclosure is negative.

Sample size	Porcine embryo gonads were obtained from five pregnant sows.
Data exclusions	NO exclusion.
Replication	Experimental analyses were conducted with at least three independent replicates.
Randomization	In this study, there were no clinical populations or patients, therefore randomization techniques were not applicable.
Blinding	Blinding is not applicable to the current work since clinical populations or patients were not involved.

Reporting for specific materials, systems and methods

We require information from authors about some types of materials, experimental systems and methods used in many studies. Here, indicate whether each material, system or method listed is relevant to your study. If you are not sure if a list item applies to your research, read the appropriate section before selecting a response.

Materials & experimental systems

n/a	Involved in the study
☐	☒ Antibodies
☒	☐ Eukaryotic cell lines
☒	☐ Palaeontology and archaeology
☐	☒ Animals and other organisms
☒	☐ Clinical data
☒	☐ Dual use research of concern
☒	☐ Plants

Methods

n/a	Involved in the study
☒	☐ ChIP-seq
☒	☐ Flow cytometry
☒	☐ MRI-based neuroimaging

Antibodies

Antibodies used

Rat polyclonal antiBLIMP1(Thermo Fisher Scientific, Cat#14-5963-80, dilution 1:300);
Rabbit monoclonal anti-DAZL(abcam, Cat#ab215718, dilution 1:300);
Rabbit polyclonal anti-DDX4(abcam, Cat#ab13840, dilution 1:200);
Mouse monoclonal anti-γH2AX(abcam, Cat#ab26350, dilution 1:200);
Mouse monoclonal anti-5mC(Active Motif, Cat#39649, dilution 1:500);
Rabbit polyclonal anti-5hmC(Active Motif, Cat#39791, dilution 1:500);
Rabbit polyclonal anti-H3K27me3(Active Motif, Cat# 39155, dilution 1:300);
Rabbit polyclonal anti-KRT19(Proteintech, Cat# 10712-1-AP, dilution 1:200);
Rabbit polyclonal anti-WT1(Proteintech, Cat# 12609-1-AP, dilution 1:200);
Mouse monoclonal anti-GATA4(Santa Cruz Biotechnology, Cat# sc-25310, dilution 1:300);
Rabbit polyclonal anti-CD31(abcam, Cat# ab28364, dilution 1:300);
Rabbit polyclonal anti-LAMA1(Sigma-Aldrich, Cat# L9393, dilution 1:500);
Rabbit polyclonal anti-PDGFRA(Absin, Cat# abs146265, dilution1:200);
Rabbit polyclonal anti-CTNNB1(abcam, Cat# ab6302, dilution 1:300);
Rabbit polyclonal anti-Phospho-SMAD1/5(Cell Signaling Technology, Cat#9516T, dilution 1:100);
Rabbit polyclonal anti-c-KIT(abcam, Cat#ab32363, dilution 1:300);
RNAscope Probe -Ss-LHX9-C1(ACDBio, Cat#1807211-C1);
RNAscope Probe -Ss-LAMA1-C2(ACDBio, Cat#1807221-C2) ;
RNAscope Probe -Ss-MAN1A1-C3(ACDBio, Cat#1807231-C3);
RNAscope Probe -Ss-RARB-C2(ACDBio, Cat#1807241-C2);
TSA Vivid Fluorophore 520 (ACDBio, Cat#PG-323271);
TSA Vivid Fluorophore 570 (ACDBio, Cat#PG-323272);
TSA Vivid Fluorophore 650 (ACDBio, Cat# PG-323273);
Donkey anti-Rat IgG (H+L) Highly Cross-Adsorbed Secondary Antibody, Alexa Fluor 488(Thermo Fisher Scientific, Cat#A-21208, dilution 1:1000);
Donkey anti-Mouse IgG (H+L) Highly Cross-Adsorbed Secondary Antibody, Alexa Fluor 488(Thermo Fisher Scientific, Cat#A-21202, dilution 1:1000);
Donkey anti-Mouse IgG (H+L) Highly Cross-Adsorbed Secondary Antibody, Alexa Fluor 594(Thermo Fisher Scientific, Cat#A-21203, dilution 1:1000);
Donkey anti-Rabbit IgG (H+L) Highly Cross-Adsorbed Secondary Antibody, Alexa Fluor 488(Thermo Fisher Scientific, Cat#A-21206, dilution 1:1000);
Donkey anti-Rabbit IgG (H+L) Highly Cross-Adsorbed Secondary Antibody, Alexa Fluor 594(Thermo Fisher Scientific, Cat# A-21207, dilution 1:1000);
Rat polyclonal antiBLIMP1(Thermo Fisher Scientific, Cat#14-5963-80, dilution 1:300);
Rabbit monoclonal anti-DAZL(abcam, Cat#ab215718, dilution 1:300);
Rabbit polyclonal anti-DDX4(abcam, Cat#ab13840, dilution 1:200);
Mouse monoclonal anti-γH2AX(abcam, Cat#ab26350, dilution 1:200);
Mouse monoclonal anti-5mC(Active Motif, Cat#39649, dilution 1:500);
Rabbit polyclonal anti-5hmC(Active Motif, Cat#39791, dilution 1:500);
Rabbit polyclonal anti-H3K27me3(Active Motif, Cat# 39155, dilution 1:300);
Rabbit polyclonal anti-KRT19(Proteintech, Cat# 10712-1-AP, dilution 1:200);
Rabbit polyclonal anti-WT1(Proteintech, Cat# 12609-1-AP, dilution 1:200);
Mouse monoclonal anti-GATA4(Santa Cruz Biotechnology, Cat# sc-25310, dilution 1:300);
Rabbit polyclonal anti-CD31(abcam, Cat# ab28364, dilution 1:300);
Rabbit polyclonal anti-LAMA1(Sigma-Aldrich, Cat# L9393, dilution 1:500);
Rabbit polyclonal anti-PDGFRA(Absin, Cat# abs146265, dilution1:200);
Rabbit polyclonal anti-CTNNB1(abcam, Cat# ab6302, dilution 1:300);
Rabbit polyclonal anti-Phospho-SMAD1/5(Cell Signaling Technology, Cat#9516T, dilution 1:100);
Rabbit polyclonal anti-c-KIT(abcam, Cat#ab32363, dilution 1:300);
RNAscope Probe -Ss-LHX9-C1(ACDBio, Cat#1807211-C1);
RNAscope Probe -Ss-LAMA1-C2(ACDBio, Cat#1807221-C2) ;
RNAscope Probe -Ss-MAN1A1-C3(ACDBio, Cat#1807231-C3);
RNAscope Probe -Ss-RARB-C2(ACDBio, Cat#1807241-C2);
TSA Vivid Fluorophore 520 (ACDBio, Cat#PG-323271);
TSA Vivid Fluorophore 570 (ACDBio, Cat#PG-323272);
TSA Vivid Fluorophore 650 (ACDBio, Cat# PG-323273);
Donkey anti-Rat IgG (H+L) Highly Cross-Adsorbed Secondary Antibody, Alexa Fluor 488(Thermo Fisher Scientific, Cat#A-21208, dilution 1:1000);

	Donkey anti-Mouse IgG (H+L) Highly Cross-Adsorbed Secondary Antibody, Alexa Fluor 488(Thermo Fisher Scientific, Cat#A-21202, dilution 1:1000);<br>Donkey anti-Mouse IgG (H+L) Highly Cross-Adsorbed Secondary Antibody, Alexa Fluor 594(Thermo Fisher Scientific, Cat#A-21203, dilution 1:1000);<br>Donkey anti-Rabbit IgG (H+L) Highly Cross-Adsorbed Secondary Antibody, Alexa Fluor 488(Thermo Fisher Scientific, Cat#A-21206, dilution 1:1000);<br>Donkey anti-Rabbit IgG (H+L) Highly Cross-Adsorbed Secondary Antibody, Alexa Fluor 594(Thermo Fisher Scientific, Cat# A-21207, dilution 1:1000);
Validation	Rat polyclonal anti-BLIMP1(https://www.thermofisher.cn/cn/zh/antibody/product/Blimp-1-Antibody-clone-6D3-Monoclonal/14-5963-82)<br>Rabbit monoclonal anti-DAZL(https://www.abcam.cn/products/primary-antibodies/dazl-antibody-epr21028-ab215718.html);<br>Rabbit polyclonal anti-DDX4(https://www.abcam.cn/products/primary-antibodies/ddx4--mvh-antibody-ab13840.html);<br>Mouse monoclonal anti-γH2AX(https://www.abcam.cn/products/primary-antibodies/gamma-h2ax-phospho-s139-antibody-9f3-ab26350.html);<br>Mouse monoclonal anti-5mC(https://www.activemotif.com/catalog/details/39649/5-methylcytosine-5-mc-antibody-mab-clone-33d3);<br>Rabbit polyclonal anti-5hmC(https://www.activemotif.com/catalog/details/39791/5-hydroxymethylcytidine-antibody-pab);<br>Rabbit polyclonal anti-H3K27me3(https://www.activemotif.com/catalog/details/39155/histone-h3-trimethyl-lys27-antibody-pab);<br>Rabbit polyclonal anti-KRT19(https://www.ptgcn.com/results?category=&filter=&q=10712-1-AP);<br>Rabbit polyclonal anti-WT1(https://www.ptgcn.com/results?category=&filter=&q=12609-1-AP);<br>Mouse monoclonal anti-GATA4(https://www.scbt.com/zh/p/gata-4-antibody-g-4);<br>Rabbit polyclonal anti-CD31(https://www.abcam.cn/products/primary-antibodies/cd31-antibody-ab28364.html);<br>Rabbit polyclonal anti-LAMA1(https://www.sigmaaldrich.cn/CN/zh/product/sigma/l9393);<br>Rabbit polyclonal anti-PDGFRA(https://www.absin.cn/anti-pdgfr-alpha-antibody/abs146265.html);<br>Rabbit polyclonal anti-CTNNB1(https://www.abcam.cn/products/primary-antibodies/beta-catenin-antibody-ab6302.html);<br>Rabbit polyclonal anti-Phospho-SMAD1/5(https://www.cellsignal.com/products/primary-antibodies/phospho-smad1-5-ser463-465-41d10-rabbit-mab/9516);<br>Rabbit polyclonal anti-c-KIT(https://www.abcam.cn/products/primary-antibodies/c-kit-antibody-yr145-ab32363.html);<br>RNAscope Probe -Ss-LHX9-C1(https://www.bio-techne.com/cn/p/in-situ-hybridization-probes/rnascope-hiplex-probe-ss-lhx9_1807211-t1);<br>RNAscope Probe -Ss-LAMA1-C2(https://www.bio-techne.com/cn/p/in-situ-hybridization-probes/rnascope-hiplex-probe-ss-lama1_1807221-t1) ;<br>RNAscope Probe -Ss-MAN1A1-C3(https://www.bio-techne.com/cn/p/in-situ-hybridization-probes/rnascope-hiplex-probe-ss-man1a1_1807231-t1);<br>RNAscope Probe -Ss-RARB-C2(https://www.bio-techne.com/cn/p/in-situ-hybridization-probes/rnascope-hiplex-probe-ss-rarb_1807241-t1);<br>TSA Vivid Fluorophore 520 (https://www.bio-techne.com/cn/p/in-situ-hybridization-ancillaries/tsa-vivid-fluorophore-520_323271);<br>TSA Vivid Fluorophore 570 (https://www.bio-techne.com/cn/p/in-situ-hybridization-ancillaries/tsa-vivid-fluorophore-570_323272);<br>TSA Vivid Fluorophore 650 (https://www.bio-techne.com/cn/p/in-situ-hybridization-ancillaries/tsa-vivid-fluorophore-650_323273);<br>Donkey anti-Rat IgG (H+L) Highly Cross-Adsorbed Secondary Antibody, Alexa Fluor 488(https://www.thermofisher.cn/cn/zh/antibody/product/Donkey-anti-Rat-IgG-H-L-Highly-Cross-Adsorbed-Secondary-Antibody-Polyclonal/A-21208);<br>Donkey anti-Mouse IgG (H+L) Highly Cross-Adsorbed Secondary Antibody, Alexa Fluor 488(https://www.thermofisher.cn/cn/zh/antibody/product/Donkey-anti-Mouse-IgG-H-L-Highly-Cross-Adsorbed-Secondary-Antibody-Polyclonal/A-21202);<br>Donkey anti-Mouse IgG (H+L) Highly Cross-Adsorbed Secondary Antibody, Alexa Fluor 594(https://www.thermofisher.cn/cn/zh/antibody/product/Donkey-anti-Mouse-IgG-H-L-Highly-Cross-Adsorbed-Secondary-Antibody-Polyclonal/A-21203);<br>Donkey anti-Rabbit IgG (H+L) Highly Cross-Adsorbed Secondary Antibody, Alexa Fluor 488(https://www.thermofisher.cn/cn/zh/antibody/product/Donkey-anti-Rabbit-IgG-H-L-Highly-Cross-Adsorbed-Secondary-Antibody-Polyclonal/A-21206);<br>Donkey anti-Rabbit IgG (H+L) Highly Cross-Adsorbed Secondary Antibody, Alexa Fluor 594(https://www.thermofisher.cn/cn/zh/antibody/product/Donkey-anti-Rabbit-IgG-H-L-Highly-Cross-Adsorbed-Secondary-Antibody-Polyclonal/A-21207);

Animals and other research organisms

Policy information about studies involving animals; ARRIVE guidelines recommended for reporting animal research, and Sex and Gender in Research

Laboratory animals	Porcine embryos were obtained from pregnant sows (Large White) via artificial insemination at local breeding facilities. Fetal gonads were harvested at 24, 27, 30, 35, and 50 days post-fertilization (E24–E50).
Wild animals	No wild animals were used in this study.
Reporting on sex	This study investigates the process of oogenesis, utilizing female embryos for subsequent research. The sex of embryos was determined by sex determination PCR(pig SRY F: GGGAAAGGCTCCTCACTATTT,R: AGGGATACATCCTCTCCTCTAC; pig ZFX F: GTGCTGCTTTGTCTTGGAATG,R: GAGGGAGTTAGGTCTGGATACT)
Field-collected samples	No field collected samples were used in the study.
Ethics oversight	All of the pig experiments performed were approved by the Institutional Animal Care and Use Committee of China Agricultural University.

Note that full information on the approval of the study protocol must also be provided in the manuscript.

Plants

Seed stocks	N/A
Novel plant genotypes	N/A
Authentication	N/A
